# Optimizing college health promotion in the digital age: Comparing perceived well-being, health behaviors, health education needs and preferences between college students enrolled in fully online versus campus-based programs

**DOI:** 10.15171/hpp.2019.37

**Published:** 2019-10-24

**Authors:** Michelle M. Burcin, Shelley N. Armstrong, Jody O. Early, Holly Godwin

**Affiliations:** ^1^Walden University School of Health Sciences, Minneapolis, MN, USA; ^2^University of Washington Bothell, School of Nursing and Health Studies, Bothell, WA, USA

**Keywords:** Online students, Health behavior, College students, College wellness, Digital health

## Abstract

**Background:** There is little published about non-traditional and online college students’ health and well-being. College health services must evolve to address the needs of this growing population. The purpose of this study was to explore risk factors, perceived well-being, health behaviors, and health education preferences of US college students enrolled in a fully online academic programs compared to a national sample of college students enrolled in campus based programs.

** Methods:** This cross-sectional study included a volunteer sample of 961 college students enrolled in two large, U.S. accredited online universities. Participants completed an online survey that included questions and sub scales from the National College Health Assessment (NCHA, IIb). Responses on survey items from student learning online were compared to an equal sample of college students enrolled in non-online programs, randomly drawn from the NCHA IIb national data set (n = 961). Frequencies on survey items were calculated and mean scores of subset measures for online students were compared against those from the NCHA data set using two tailed z-test scores and independent sample t-tests with alpha at 0.05.

**Results:** Online students reported significantly (P ≤ 0.05) higher percentages of chronic illnesses, psychiatric conditions, mobility disabilities, deafness/hearing loss, speech/language disorders,cigarette use, obesity, sedentary activity, and depression than the NCHA national sample.

**Implication for Practice: **Health professionals and leaders who work in higher education must consider the shifting landscape and demographics in higher education in order to develop more tailored, innovative digital health promotion approaches that effectively reach the growing population of online, commuter, and older learners.

## Background

Enrollment in online university programs have increased over the last ten years,^1^ yet the health risks and needs of online learners are not well understood. A few studies have made conclusions about the online learning environment and the students’ being at higher risk of sedentary lifestyles, cigarette smoking, and depression due to social isolation.^2-4^ Rohrer et al found that nearly a quarter of online student respondents were smokers and may favor the online learning environment because studying at home permits them to smoke.^3^ However, a recent exploratory study suggested that being an online student was not a risk factor for poor health.^5^ The results of these studies are inconsistent and contain many limitations, particularly the use of non-random sampling which may make the sample not representative of the online student population.^3,5^ In addition, when online students are surveyed using a snowball sampling method,^3^ it is not possible to benchmark them to students nationwide.

On the contrary, college students enrolled in campus-based programs health risks and needs are well understood. The American College Health Association (ACHA) is the leading organization with the goal of “improving the health of the nation’s college students through social justice, student-centered services, professional excellence and responsiveness, multidisciplinary and collaborative approaches to health, active involvement of students, and evidence-informed program practices.”^6^ The ACHA’s National College Health Assessment II (ACHA-NCHA II) is a national research survey which assists college health service providers, health educators, counselors, and administrators in collecting data about their students’ habits, behaviors, and perceptions on the most prevalent health topics. The ACHA-NCHA provides the largest known comprehensive data set on the health of college students, providing the college health and higher education fields with a vast spectrum of information on student health.^7^ The ACHA-NCHA II includes questions related to demographics, overall health status, alcohol, tobacco, weight, nutrition, exercise, mental health, and physical health. Results from the Spring 2015 NCHA indicated that anxiety, sleep difficulties, and stress were the most commonly reported health impediments to academic performance.^7^ In addition, 35% of respondents reported feeling so depressed it was difficult to function in the last 12 months, with over 50% reporting overwhelming anxiety and over 60% reporting feeling very sad in the last 12 months.^7^However, only 15% of students experiencing anxiety and 13% of those experiencing depression reported being treated or diagnosed by a professional within the last 12 months. Interestingly, 92% of respondents reported being full-time students, with a median age of 21 years, and only 14% indicated that outside work impacted their academic performance.^7^ Thus, this sample may be vastly different in terms of employment status and health challenges experienced by students enrolled in fully, online programs. With comparative assessment data of online students, *Healthy Campus* initiatives can shift to align with the needs and characteristics of this population.

## Purpose

The purpose of this study was to explore risk factors, perceived well-being, health behaviors, and health education preferences between US college students enrolled in a fully online academic programs compared to a national sample of college students enrolled in campus-based programs.

According to an annual survey of online college students in the United States by Clinefelter and Aslanian,^8^ an undergraduate online student in the United States in 2016 was most likely a woman in her 30s, in a relationship, and caring for at least one child while working full-time.^8^ Given the demographic differences between online students and their peers at brick and mortar institutions, our hypothesis was that we would see significant differences between the two groups in terms in of health habits, behaviors, and perceptions. Through qualitative (open-ended questions), we also explored students’ perceived impediments to academic performance and their health education needs and preferences.

## Materials and Methods

### Sample

A volunteer sample of 961 college students enrolled in two large, accredited, fully online universities in the United States comprised the online student sample. At university number one, students were invited to participate through the school’s Participant Pool, a virtual bulletin board that connects researchers to participants. At university number two, students were recruited through the school’s “eCampus” portal, where students access their classrooms and support services. The announcement sent through communication listservs at both institutions invited students to take part in the research study and provided an explanation of the study aims. Participants were eligible to participate if they were currently enrolled in a fully online program and were 18 years of age or older. The study was hosted online in SurveyMonkey. The first page of the online survey was the consent form. It included background information, the voluntary nature of the study, risks and benefits, confidentiality, contacts and questions about rights as a participant, and the statement of consent via an electronic signature, regulated by the Uniform Electronic Transactions Act. Participants were informed that the maximum time commitment to complete the survey was 20 minutes, there was no monetary benefit, participation was optional and they could opt out at any time.

The NCHA archive and database in Spring 2015 included 91 497 completed college surveys and provided data for a comparison group. The comparison group completed the NCHA in the Spring of 2015 via an online surveying tool, Qualtrics. The comparison group received the survey link via a direct email from ACHA. The data was provided with permission by the ACHA.

### Instrument

After electronically signing the consent form, participants completed a 36-item online survey comprised of subscales from the ACHA’s NCHA IIb. The NCHA is the most widely recognized survey used in college health in the United States.^7^ The original NCHA was developed by an interdisciplinary team of college health professionals and was pilot tested in 1998-1999 and has since undergone test for reliability and validity^9^ comparing common survey items with national surveys such as the National College Health Risk Behavior Survey (from the Centers for Disease Control) and the College Alcohol Study by the Harvard School of Public Health. The survey consists of 294 questions regarding physical and mental health, health education, and alcohol, tobacco, and drug use, as well as questions about impediments to academic performance and risk factors. Although the NCHA is not without limitations, a number of major studies has shown evidence that the ACHA-NCHA has “empirical value for representing the nation’s students.”^7^ The Spring 2015 data set included a sample of over 108 US colleges and universities and a total of 93 034 participants.

Permission was granted by ACHA to use select subscales of the NCHA IIb in order to create the 36-item online survey for online learners. The entire NCHA IIb was not utilized for this target population because some items, such as contraceptive use and frequency of “partying”, needed to be excluded due to a high rate of “not applicable” responses among the more “non-traditional” adult population. The researchers also wanted to keep the survey short and focused on issues that would be appropriate for addressing health promotion programming and services at the university level.

In order to better align with health risk factors and issues reported to be of influence to the well-being of non-traditional, online learners, the subscales included questions relating to: demographics (11 questions), health status and chronic health problems (CHP; 2 questions), alcohol and tobacco use (ATU; 2 questions), weight status, nutrition, exercise (WSNE; 7 questions), mental health (MH; 7 questions), academic impediments (AI; 1 question), and physical health (PH; 2 questions). Three additional questions were added about health education topic, modality and impact specifically “do you think your health and well-being impacts your academic standing?”, “are you interested in receiving information on the following topics”, and “if virtual services were offered, how would you like to receive this information?” Reliability analysis reported by the ACHA in 2013 demonstrated primarily moderate to strong scores for NCHA IIb sub scale items with reported standardized alphas as follows: 0.59 (CHP), 0.74 (ATU), 0.86 (WSNE), 0.84 (MH), 0.88 (AI), and 0.83 (PH).^9^

### Statistical analyses

Descriptive and inferential statistical tests were used to analyze the data. The volunteer, online student sample was first screened for outliers, incomplete surveys, and participants that did not meet the study criteria. Forty-seven responses were excluded to arrive at the final total of 961. Frequencies on survey items were calculated and mean scores of subset measures for online students were compared against the NCHA IIb Spring 2015 data set using two-tailed z-test scores and independent sample *t* tests with alpha at 0.05. Online software Medcalc.com was used to calculate the *t* tests on the means and Social Science Statistics (online) was used for all z-calculations on the proportions.

## Results

### Demographic characteristics of the samples

There were notable differences of demographic characteristics between the study sample (e.g. online students) and the comparison group (NCHA group) (Table 1). Of the 961 fully online students who completed the survey, the mean age was 40.2 years compared to 22.6 years for the NCHA group. The difference in the means of 17.6 years was highly statistically significant (*P* value < 0.0001). The student samples also differed in representation by racial/ethnic breakdown. More students identified as African American/Black in the online student sample (28.6%) compared to the NCHA group (5.5%) and more Hispanic/Latino students in the NCHA group than the online student sample (11.2% versus 5.2%). In the online student sample 2.6% identified as Asian/Pacific Islander compared to 13.7% in the NCHA sample and approximately 4% reported they were bi- or multi-racial in both samples.

The majority of students in the online and NCHA samples identified as female, 70.2% and 66.6% respectively. In terms of their sexual identity, the majority of students in both groups identified as heterosexual (90.0% for online students and 88.5% of non-online students), with the remainder identifying as LGBTQIA (9.1% online students vs. 11.5% non-online students).

More online students reported being married or partnered (53.9%) compared to students in the NCHA sample (9.3%), which is assumed to be based differences in age. The majority of online students worked 40 or more hours per week (61.8%) compared to the NCHA sample (6.5%), a highly statistically significant result (t-statistic = 92.93, *P* value < 0.0001). In addition, 92.0% students in the NCHA sample reported they were in school full-time versus 72.7% of students in fully online programs.

### Health status and chronic health problems reported by online students vs. NCHA group

***A*** higher percent of online students (15.7%) reported having been diagnosed with a chronic illness and/or disability (e.g. cancer, diabetes, autoimmune disorders) compared to 4.9% of the NCHA group (Table 2). Online college students were more likely to report a psychiatric condition (11.3%) compared to those in the NCHA sample (7.1%). The online sample also reported a higher percentage of a mobility disability (7.1%) versus the NCHA data set (0.9%). attention deficit/hyperactivity disorder (ADHD) was the highest reported health issue among the NCHA sample (7.4%) which is still lower than online student sample reporting ADHD (7.9%). More than double the percent of students in the online sample report deafness or problems with sight (10.1%) versus the NCHA sample (4.2%). The percent of students reporting a learning disability for both groups is around 4%. Test statistics for chronic illness (*z* = 14.90, *P*value < 0.0001), deafness/hearing loss (*z* = 8.16, *P*value < 0.0001), psychiatric condition (*z* = 4.98, *P*value < 0.0001), and speech or language disorder (*z* = 3.79, *P*value < 0.0001) were all statistically significant. The tests for learning disability (*z* = 0.55, *P*value = 0.58) and attention deficit disorder (*z* = 0.56, *P*value = 0.58) were not significant and we failed to reject that the null hypothesis was true.

### Alcohol and tobacco use

The NCHA sample had statistically significantly higher percentages of students binge drinking (e.g. consuming 5 or more drinks in one sitting) over the previous 2 weeks compared to the online college students.

Although a greater percentage of online students in the sample (8.1%) reported smoking daily versus the NCHA sample (2.5%) (*z* = 10.57, *P*value < 0.0001), cigarette use in the 30 days prior to completing the survey is higher in the NCHA sample at 8.0% compared to 4.7% of the online students (Table 3). Daily use of cigars, little cigars, clove cigarettes, and smokeless tobacco were similar for both groups.

### Weight status, nutrition and exercise

There were statistically significant differences between the two groups on weight status (Table 4). Over 66% of online students classified themselves as overweight (*z* = 8.73, *P*value < 0.0001) to very overweight (*z* = 22.67, *P*value < 0.0001) with 69.4% currently trying to lose weight (*z* = 10.47, *P*value < 0.0001), compared to the NCHA sample with 36.4% classifying themselves as overweight to very overweight and 52% trying to lose weight. The percentage of online students who consumed three or more servings of fruits and vegetables per day was higher (40.2%) compared to the NCHA sample (33.1%), although more than half in both samples reported consuming two or fewer servings per day. Regarding exercise, online students had a higher percent of zero days per week of: moderate-intensity cardio (30.5%), vigorous intensity (53.5%) and strength training (56.5%). Only 15.5% reported engaging in five or more days each week. A greater percentage of online students (53.5%) reported no days of vigorous-intensity cardio per week, compared to 41.1% of the NCHA sample. Only 8.6% of online students reported engaging in five or more days each week of vigorous cardio. Additionally, 56.6% of online students reported no (0 days) of strength training per week. Finally, 61.3% of online students reported sitting more than 6 hours each day.

### Mental health

Regarding mental health, the survey inquired about students’ feelings in the previous two weeks. The NCHA sample had statistically significant higher proportions for: “Overwhelmed by all I have to do” (*z* = -9.12, *P*value < 0.0001); “felt very lonely” (*z* = -7.98, *P*value < 0.0001); “felt very sad” (*z* = -6.53, *P*value < 0.0001); “felt so depressed it was difficult to function” (*z* = -3.27, *P*value < 0.0001); and “felt overwhelming anxiety” (*z* = -2.51, *P*value < 0.0001) (Table 5). However, a higher percentage of online students (29.3%) reported having been diagnosed with depression (*z* = 7.85, *P*value < 0.0001) compared to the NCHA sample (19.1%). For both samples, more than half reported experiencing “more than average” to “tremendous levels” of stress over the previous 12 months (online= 51.9%, NCHA= 53.2%). Regarding sleep, the NCHA sample had a statistically significant higher percent of students who reported not getting enough rest to feel sleepy during the day (41.5%) for “more than a little problem” to a “very big problem” (*z* = -6.39, *P*value < 0.0001) compared to 31.1% of the online student sample.

### Academic impediments

The top five health impediments to academic performance reported by online students were: 1) stress; 2) chronic health problem; 3) work; 4) anxiety; and 5) death of a family/friend (Table 6). The top five health education topics of interest reported by online college students were: 1) stress; 2) nutrition; 3) physical activity; 4) sleep difficulties; 5) how to help others in distress.Stress, anxiety, and work ranked as top academic impediments for both groups, but chronic health problems and death of a family/friend were specific to online students. The majority (71%) of online students believed that health and well-being impacted their academic standing, and 70.1% indicated “yes” they would participate in virtual health services and use electronic health promotion resources or programs if they were offered at their university.

## Discussion

There are very few published studies which examine the health of online college students compared to students enrolled in brick-and-mortar academic programs. This study compared health habits, behaviors, and perceptions of online students attending two large, accredited US universities (n = 961) to an equal comparison group of non-online students drawn from the 2015 NCHA IIb dataset. Significant differences in age, race/ethnicity, marital status and hours worked were identified between the two groups. Online students in this study were older (40 years). More participants identified as African American/Black and Hispanic/Latino; more were married or divorced; and the majority worked 40 or more hours per week. The sample characteristics of the study are consistent with the demographic breakdown of a what is characterized in the literature as “non-traditional” student population: 57% female, 34 years being the average age, almost 30% identifying as non-Caucasian, and the majority working full or part-time (60% for undergraduates and 88% for graduate students).^8^ The demographics are also more consistent with community college students.^10^ As non-traditional students have become the majority (71%) on college campuses,^11^ the term “non-traditional” is no longer fitting. This demographic shift should inform the college and university *Healthy Campus* initiatives as well as health promotion strategies and services.

The results of this study also demonstrated that online students had significantly higher percentages of having chronic illnesses, psychiatric conditions, mobility disabilities, deafness/hearing loss, and/or speech or language disorders versus the college students from the NCHA data set. These findings are consistent with the literature that notes online students are vulnerable to challenges affecting their well-being, levels of stress and satisfaction, academic persistence and diploma attainment.^11^

In addition, online students had statistically significant higher percentages of daily cigarette as well as higher percentages of students classifying themselves as “overweight” to “very overweight” when compared to the college students from the NCHA data set (66.5% versus 36.4%, respectively). Yet 52% of college students from the NCHA data set reported trying to lose weight compared to 69.4% of online students. Therefore, online students may have a more realistic perception of body image when compared to the NCHA sample. This clearly aligns with research studies that have demonstrated that symptoms of eating disorders are pervasive among college students, with a prevalence ranging from 8% to 17%.^12-14^ This may be due to structural and psychosocial influences, such as stress, lower levels of self-esteem and sense of unity.^15^

Obesity is a major health problem in the United States, affecting roughly one-third of adults, and is associated with multiple chronic health problems.^16^ Lack of exercise is the largest contributor to obesity in America. In relation to this, online students had a higher percent of sedentary activity and these findings are consistent with the literature concludingonline students’ are at higher risk of sedentary lifestyles and cigarette smoking.^3^ Researchers also reported older students having significantly higher body mass index (BMI) scores, and smoking being associated with higher BMI.^17^ The demographics of this sample of online students (being older, married, employed, more ethnically diverse) are known to negatively influence physical activity behavior.^18^ Furthermore, screen time (amount of time spent in front of a television, computer, or other electronic devices) has been linked to obesity.^19^ Several studies have concluded that a higher screen time (more than 21 hours per week) was associated with obesity, regardless of exercise behavior.^17,19,20^

Online students had statistically significant lower proportions for being overwhelmed, lonely, sad, depressed, and anxiety over the past two weeks, but a statistically significant higher percentage of having been diagnosed with depression. More information and research is needed in this area, but one could speculate that online students are older and therefore have found coping skills and techniques to deal with depressive symptoms. Stress was the top health impediment to academic performance reported by both groups. According to a recent study, mental health and stress management were the most common types of health services offered by universities offering fully online academic programs.^21^ However, services were offered in the form of written material or website. The results from this study also underscored the need for mental health services promoted to online students to be more interactive and possibly synchronous. For example, support groups and coaching might be offered virtually. Handouts and websites provide a good starting point, but they do not create a healthy campus climate, offer behavior change techniques, or support for real health actualization.^21^

In terms of health education needs and preferences, the top five health education topics of interest to online college students only slightly differed from those in the non-online group. For the online group, the top issues of need or interest were: 1) stress; 2) nutrition; 3) physical activity; 4) sleep difficulties; 5) how to help others in distress. Topics reported by non-online students included the following: 1) mental health; 2) stress management; 3) physical activity/fitness; 4) relationship violence/sexual assault; and 5) weight management.^21^

### Limitations

This study, like all research, was not without limitations. The first was that participants in the online group were selected through convenience sampling of two fully online universities and did not reflect all online students in the United States. This may have limited variation in responses and thereby inhibits generalizability of findings. In addition, the NCHA IIb subscales used to collect the data only provide a snapshot of a college student’s health and well-being, as defined by the limitations of the scale and self-reported data. Although it is the most widely used college health instrument, the NCHA has a number of limitations such as item sensitivity, not defining health behaviors, and absence of theory, as recently outlined by researchers Rahn, Pruitt and Goodson.^22^ Ideally, future iterations of the NCHA would account for the shifting demographics in higher education and the fact that more full-time students are enrolling in fully online academic programs.

Also, future research on fully online students’ health issues should take into consideration the following variables: age, identified gender (to include non-binary), work status, timing of survey, type of campus, and major life events that may influence answers. Additionally, to improve generalizability, future studies should use a national sample of online students and compare this to a sample of non-online students of similar age and work profiles. Although similar differences could be found between two groups within the general population with such different demographics, this study highlights the changes colleges and universities have to promote health and well-being of their online students.

### Implications for Practice

Assessing community capacity and needs, developing tailored health education and promotion programs and services, and delivering health education in formats most likely to reach the intended audience are primary responsibilities of health education specialists.^23^ Although wellness data is collected yearly across college campuses in the United States, there has previously been little known about online students’ health needs and behaviors, or how college health services are evolving, to address the needs of this population.

The study findings underscored the need for US colleges and universities to increase digital health promotion resources, programs, and strategies tailored for the growing number of online, place-bound, and commuter students. The demand is there; the large majority (70.1%) of online students (n = 961) in this study indicated they would use health promotion resources and services if they were offered at their institution. However, few universities are doing more than providing written material online.^21^ In order to achieve social change and become a bigger part of the healthcare solution, university health services should positivity impact their online community’s behaviors and well-being, which requires interaction with each other, participation, leadership, and the uptake of interventions.^13^

Most of the health issues reported by students in this study are addressed by *Healthy Campus* (e.g. mental health issues such as stress, nutrition, sleep, physical activity). Stress, anxiety, and work were ranked as top academic impediments for both groups, but chronic health programs and death of a family/friend were specific to online students. These findings suggest that online students may benefit from stress, time management, and physical activity programs aimed at working adults-especially those prone to long periods of sitting.

Leveraging technology to improve college health promotion will not only cater to the preferences of more digital savvy Millennial and post-Millennial students, but will also address a resource gap, especially for those enrolled in fully online programs. Furthermore, as the results of the student survey confirmed, health and well-being are connected to academic success. By providing services and programs that address some of the most pressing health challenges, universities may simultaneously strengthen students’ academic performance and persistence.

## Ethical approval

The Institutional Review Boards (IRBs) and Institutional Approvers (IA) from both universities approved the study.

## Competing interests

The authors declare that they have no competing interests. The contents of this publication are solely the responsibility of the authors and do not necessary represent the official views of Walden University or the ACHA.

## Funding

This project was supported by Walden University via an internal faculty grant.

## Authors’ contributions

MMB and SNA secured the grant funding through Walden University to conduct this study. All authors contributed to planning and write-up of the paper. MMB and SNA implemented the study and lead the framework for the paper. All authors contributed to the data analysis, results, discussion, and conclusion. JOE helped to write the background, methods, and results and also contributed to the discussion. She also assisted with revisions. HG also contributed to the background and writing the conclusion.

## Acknowledgments

The authors would like to acknowledge the universities and students who participated in this project as well as the ACHA for allowing the authors to use a subset of questions from the NCHA.

**Table 1:** Comparison of demographic characteristics of online vs. non-online college students

	**Online College Students**	**Non-online College Students (NCHA II Spring 2015 data set)**	**Z-Statistic**	***P*** **Value**
Race/ethnicity	n = 961	n = 103 314		
White/Caucasian	55.30%	60.90%	3.54	0.0004
Black/African American	28.60%	5.50%	-30.71	<0.0001
Hispanic/Latino	5.20%	11.20%	5.88	<0.0001
Asian/Pacific Islander	2.60%	13.70%	9.99	<0.0001
Bi/Multi-racial	4.50%	4.10%	-0.62	0.5353
Age [mean]	n = 961	n = 91 966		
	40.2 years	22.6 years	-91.44	<0.0001
Gender	n = 961	n = 93 034		
Female	70.24%	66.6%	-2.4	0.164
Male	29.45%	32.19%	1.81	0.0703
Transgender	0.31%	0.45%	8.68	<0.0001
Enrollment status	n = 961	n = 92 272		
Full Time	72.70%	92.00%	21.7	<0.0001
Part Time	24.80%	7.30%	-20.52	<0.0001
International status	n = 961	n = 92 068		
	12.20%	9.50%	-2.84	0.0045
Sexual orientation	n = 961	n = 92 065		
Heterosexual	90.90%	88.50%	-2.32	<0.0203
Relationship status	n = 961	n = 92 092		
Married/partnered	53.90%	9.30%	-46.35	<0.0001
Single	28.80%	87.90%	54.72	<0.0001
Divorced	12.10%	1.10%	-30.98	<0.0001
Hours worked per week	n = 961	n = 91 945		
0	18.00%	41.40%	-14.69	<0.0001
1-39	20.20%	52.10%	19.69	<0.0001
40	29.80%	3.40%	42.96	<0.0001
40+	32.00%	3.00%	49.97	<0.0001

**Table 2:** Comparison of chronic illness and disabilities between online vs. non-online students

	**Online College Students**	**Non-Online College Students NCHA**	**Z-Statistic**	***P*** **Value**
Attention deficit	n = 961	n = 91,829		
	7.85 %	7.37%	0.5602	0.5755
Chronic illness	n = 961	n = 91,822		
	15.70%	4.91%	14.9	<0.0001
Deafness	n = 961	n = 91,782		
	5.56%	1.86%	8.163	<0.0001
Learning disability	n = 961	n = 91,523		
	4.36%	4.00%	0.5528	0.5823
Psychiatric	n = 961	n = 91,699		
	11.34%	7.09%	4.9815	<0.0001
Speech or language	n = 961	n = 91,693		
	1.96%	0.82%	3.7861	<0.0002

**Table 3:** Frequencies of cigarette use and alcohol use: online students versus non-online students

	**Online College Students** **(n = 961)**	**Non-online College Students (NCHA II Spring 2015 data set; n = 91829)**	**Z-Statistic**	***P*** **Value**
Cigarette use				
Never used	73.4%	73.8%	-0.27	0.7872
Used last 30 days	4.7%	8.0%	-3.72*	0.0002
Smoke daily	8.1%	2.5%	10.57*	<0.0001
Daily use of cigars, little cigars, clove cigarettes	0.3%	0.1%		
Daily use of smokeless tobacco	0.7%	0.7%		
Alcohol use [5+ drinks over last 2 weeks]				
None/do not drink	29.3%	23.7%	7.59*	<0.0001
1-5 times	19.2%	29.8%	-6.99*	<0.0001
6+ times	1.0%	2.1%	-2.37*	0.0178

*Significant differences between online students and NCHA sample (*P* < 0.0001).

**Table 4:** Comparison of Weight, Nutrition and Exercise among Online vs. Non-Online College Students

	**Online College Students (n = 961)**	**Non-online College Students (NCHA II Spring 2015 data set; n = 91829)**	**Z-score**	***P*** **value**
Weight status				
Very underweight	0.9%	0.7%		
Slightly underweight	0.05%	8.9%		
About the right weight	27.7%	54.1%	-15.93*	< 0.0001
Slightly overweight	44.8%	31.4%	8.73*	< 0.0001
Very overweight	21.7%	5.0%	22.67*	< 0.0001
Trying to lose weight	69.4%	52.0%	10.47*	< 0.0001
Fruits and vegetables				
0-2 servings per day	59.8%	66.9%	-4.59*	< 0.0001
3+ servings	40.2%	33.1%	4.59*	< 0.0001
Exercise				
0 days moderate-intensity cardio	30.5%	24.6%		
5+ days moderate intensity cardio	15.5%	19.2%		
0 days vigorous cardio	53.5%	41.1%		
5+ days vigorous cardio	8.6%	10.4%		
0 days strength training	56.5%	50.7%		
5+ days strength training	7.1%	8.9%		

*Significant differences between online students and NCHA sample (*P* < 0.0001).

**Table 5:** Mental health issues reported by online college students vs. non-online college students

	**Online CollegeStudents (n = 961)**	**Non-Online College Students (NCHA II Spring 2015 data set; n = 91829)**	**Z-Statistic**	***P*** **Value**
In the last 2 weeks				
Overwhelmed by all I have to do	35.8%	51%	-9.12*	<0.0001
Felt very lonely	13.5%	25%	-7.98*	<0.0001
Felt very sad	17.0%	26.5%	-6.53*	<0.0001
Felt so depressed it was difficult to function	8.3%	11.9%	-3.37*	<0.0001
Felt overwhelming anxiety	19.5%	23.6%	-2.91*	<0.0001
Have been diagnosed with depression	29.3%	19.1%	7.85*	<0.0001
Levels of stress over last 12 months				
More than average	37.5%	42.5%	-3.25*	<0.0001
Tremendous	14.4%	10.7%	3.52*	<0.0001
Slept to feel rested in last week				
0 days	12.5%	10.7%	1.75	0.08
1-2 days	27.6%	29.9%	-1.44	0.15
3-5 days	42.9%	47.5%	-2.77*	0.01
6-7 days	16%	11.7%	4.64*	<0.0001
Felt sleepy in the daytime during the last week				
No problem	18.3%	9.9%	8.42*	<0.0001
A little problem	50.6%	48.5%		
More than a little problem	18.4%	24.9%	-6.39*	<0.0001
A big problem	9.1%	11.9%		
A very big problem	3.6%	4.7%		

*Significant differences between online students and NCHA sample (*P* < 0.0001).

**Table 6:** Academic impediments reported by online college students vs. non-online college students

**Online College Students** **(n = 961)**	**Non-Online College Students (NCHA II Spring 2015 data set)** **(n= 91819)**
1. Stress	1. Stress
2. Chronic health problem	2. Anxiety
3. Work	3. Depression
4. Anxiety	4. Sleep difficulties
5. Death of a family/friend	5. Work
